# How to explain the German East–West divide in support of a populist radical right party (AfD): mapping socioeconomic, psychological, and cultural explanatory factors from a demand-side, voter-level perspective

**DOI:** 10.3389/fsoc.2026.1759913

**Published:** 2026-05-26

**Authors:** Hermann Siebel, Gülru Horozoğlu, Ursula Kessels

**Affiliations:** Division of Heterogeneity and Education Research, Department of Education and Psychology, Freie Universität Berlin, Berlin, Germany

**Keywords:** alternative political media use, East–West divide, Germany, political distrust, populist radical right parties, populist/nativist attitudes, structural equation modeling

## Abstract

**Introduction:**

Although the success of the *Alternative für Deutschland* (AfD) is nationwide, the party received twice as many votes in East compared to West Germany in recent elections. While scholars have identified numerous general predictors of populist support, research explaining the East–West gap in AfD support remains inconclusive, largely relies on election data through 2021, and rarely integrates competing explanatory frameworks.

**Methods:**

Using representative survey data from late 2024 (*N* = 1,000), structural equation modeling was applied to make systematic, comparative claims about demand-side, voter-level explanatory factors from multiple theoretical frameworks.

**Results:**

AfD vote intention was predicted by almost all examined socioeconomic, psychological and cultural variables. Moreover, East Germans reported lower income and stronger relative deprivation, distrust of state institutions, political conspiracy mentality, use of alternative political media, populist/nativist attitudes, national identification, and importance of being German than West Germans. Yet the East–West gap in AfD vote intention was mainly accounted for by political distrust, alternative political media use, and populist/nativist attitudes, whereas socioeconomic and psychological factors did not explain the gap from a cross-sectional perspective.

**Discussion:**

This calls for further work on causally linking these frameworks to clarify demand-side mechanisms behind voting for populist radical right parties and the German East–West gap thereof.

## 1 Theoretical background

### 1.1 From founding to far right: the AfD’s evolution and ideological placement

The Alternative for Germany (“Alternative für Deutschland,” AfD) was founded in 2013 as a market-liberal, Eurosceptic political party (Arzheimer, 2015) and narrowly failed to win representation in the German parliament (“Bundestag”) in its first federal election that year (Deutscher Bundestag, 2025). Unlike other parties, its 2013 vote share differed only slightly between the former GDR states (East Germany: 5.9%) and the former FRG states (West Germany: 4.5%). Over subsequent European Parliament and federal elections, this regional gap widened incrementally (Arzheimer, 2023). In the most recent federal election on February 23, 2025, the AfD won 20.8% of the nationwide party-list (“second”) vote – its best result to date – emerging as by far the strongest party in the East with 34.5% and taking nearly twice as many votes there as in the West (17.9%, Trebing, 2025). The AfD has also performed markedly better in Eastern than in Western state elections and is currently represented in 14 of Germany’s 16 state parliaments, including all Eastern states (Henrich, 2025). Statistically, it has been repeatedly confirmed that there is a highly significant East–West gap with regard to AfD vote intention or vote choice which has widened over time (e.g., Arzheimer and Berning, 2019; Kühling et al., 2025).

Parallel to the emerging regional divergence in its electoral performance, the AfD underwent a major programmatic shift that was accompanied by a changing electorate and is ongoing until today (Kühling et al., 2025). In contrast to its beginnings (Arzheimer, 2015), the AfD is now consistently classified as part of the European family of populist radical right parties (Bieber et al., 2018; Goerres et al., 2018; Arzheimer, 2019; Arzheimer and Berning, 2019; Coury, 2021; Kühling et al., 2025). Following Mudde (2004, 2007), these parties share the core ideological features of nativism and populism despite national specialties. *Nativism*, a “thick ideology,” combines nationalism with xenophobia and depicts non-native persons and ideas as threats to an ethnically and culturally homogeneous nation-state. *Populism*, a “thin ideology,” opposes the “pure people” to a “corrupt elite” and views the democratic order as merely ruled by elites, detached from liberal institutions like courts and parliaments.

### 1.2 East versus West: a meaningful analytical category?

Especially in recent East German state election campaigns and in the most recent federal contest, one of the AfD’s core strategies – using slogans like “Wende 2.0” and “Der Osten steht auf” (“The East stands up”) – was to construct and instrumentalize a supposedly collective East German identity through a narrative of the marginalized East German (“Wendeverlierer”; “losers of the Wende”; Diermeier et al., 2024; Patton, 2019; Pickel and Pickel, 2020; Rippl, 2017; Schmalenberger, 2024; Weisskircher, 2022). Nevertheless, the programmatic shift outlined above occurred at the federal party level, and the AfD has long sought to avoid branding itself too strongly as an East German party in order to maximize its West German electoral potential (Arzheimer, 2023). For this reason, among others, Arzheimer (2021) concluded that the AfD does not meet Brancati’s (2007) criteria for regional or regionalist parties. The significant East–West gap regarding the AfD’s success can therefore not be attributed to East Germans supporting a party that is regionally distinct from its Western profile.

In recent years, scholars have started to examine which factors account for this East–West gap (Götzel, 2023). At the outset, however, it must be critically asked whether, more than three decades after reunification, the East–West divide is a somewhat artificial category with little explanatory power of its own. In line with this, some studies have searched for explanatory variables at municipal or constituency level (e.g., Bergmann et al., 2018; Deppisch et al., 2022; Nickel and Groß, 2023; Wuhs and McLaughlin, 2019), yet some of them still uncovered regional patterns that map onto the East–West divide. Even after controlling for such variables (e.g., rurality or institutional anomie), distinctively East German factors often remain – the so-called “East bonus” (Götzel, 2023). Hence, East and West Germany continue to be meaningful analytical categories, for instance when analyzing which factors account for the gap in the AfD’s electoral performance (Reiser and Reiter, 2023; Vogel et al., 2024).

### 1.3 How to explain the gap: deriving hypotheses from the theoretical frameworks

The state of research on the East–West gap in AfD support remains inconclusive (Götzel, 2023), partly because competing explanatory frameworks have rarely been integrated within the same analysis. Existing studies also largely rely on election data up to 2021; however, given the AfD’s ongoing strategic and ideological transformations and the changing broader context in which elections take place, it is important to continuously extend these valuable insights using the most recent data. Drawing on new 2024 data and using structural equation modeling (SEM), this article organizes and tests multiple explanatory frameworks at once. Using a representative sample, systematic and comparative claims about demand-side, micro-level (i.e., voter-level) factors that account for differences in stated AfD vote intention between East and West Germany are made. Therefore, in the following sections, hypotheses will be derived in accordance to three theory-driven steps, which will be repeated for each of the explanatory frameworks: First, general theories and determinants of support for populist parties (in this case, the AfD) are discussed. Second, it will be examined whether these determinants differ on average between East and West Germans.^1^ Third, as the main objective of this paper, it will be assessed whether the general determinants also specifically explain the East–West gap itself.

Since the emergence of populist radical right parties as a modern party family in the 1980s (Arzheimer, 2019), many theories and explanatory factors, driving support for such parties, have been proposed and tested (e.g., Betz, 1993). The literature on demand-side, voter-level determinants is typically structured by two broad theoretical frameworks: socioeconomic and cultural explanations (e.g., Götzel, 2023; Inglehart and Norris, 2016; Jetten and Mols, 2021; Weisskircher, 2020). In addition, individual-psychological explanations are discussed that fit neither framework (Walther and Isemann, 2019). The boundaries between these frameworks are not airtight, especially between the socioeconomic and psychological framework, which share, for example, the theory of relative deprivation. Hence, these frameworks are presented in turn. To remain within scope, the review of determinants for AfD support is limited to demand-side, voter-level explanatory factors. Supply-side (e.g., campaign funding, leadership skills) and meso-/macro-level factors (e.g., voter turnout, regional economic indicators, representation in political offices) are not taken into account. Further, the review is mostly limited to studies that use AfD support (i.e., vote intention, vote choice, or party identification) as a proxy for support of radical right-wing populism as a whole, in light of the AfD’s status as a populist radical right party (see section 1.1).

### 1.4 Socioeconomic framework

#### 1.4.1 Voting for populist parties like the AfD: socioeconomic explanations

Within the socioeconomic framework, a range of demand-side determinants is grouped under different labels, some of them stemming from supposedly distinct theories. Such labels include, for example, “relative deprivation”^2^ (operationalized via questions on whether people receive their “fair share” relative to others or via objective comparative indicators; Hartmann et al., 2022; Lux, 2018; Rippl and Seipel, 2021; Zollinger and Häusermann, 2024), “economic insecurity”/ status anxiety“(operationalized via worries about the economic future, e.g., downward mobility; Cohen, 2021; Goerres et al., 2018; Inglehart and Norris, 2016; Lengfeld and Dilger, 2018; Manow and Schwander, 2022; Rippl and Seipel, 2018; Sthamer, 2018), and general “(socio)economic deprivation” (Lengfeld, 2017; Bergmann et al., 2018; Goerres et al., 2018; Lengfeld and Dilger, 2018; Lux, 2018; Rippl and Seipel, 2018, 2021; Schmitt-Beck et al., 2019; Jetten and Mols, 2021; Hartmann et al., 2022). The latter is often operationalized via income, educational level, employment status, or other objective measures of socioeconomic status (SES). Despite the differing labels, at the voter-level, most of these explanatory factors converge on measuring a form of individual (objective or subjective) economic precarity relative to a benchmark (i.e., the perceived economic situation of others or of one’s own past, or a regional/national average).

The relationship between socioeconomic predictors and AfD support is often framed by the losers of modernization theory (Betz, 1994). This theory holds that globalization, economic restructuring and welfare retrenchment have produced “losers of modernization”, especially among low-skilled and lower-income groups in Germany and other western countries. In turn, these people should gravitate toward the AfD, not because it advocates for a strong welfare state, but because it opposes further international opening via European job market integration, promises to restrict immigration to reduce competition for scarce jobs and social benefits, and provides a vehicle for protest voting (Lengfeld, 2017). A few findings contradicted this theory (Lengfeld, 2017; Baron, 2018; Schröder, 2018; Hansen and Olsen, 2019; Hartmann et al., 2022). However, parts of this evidence have faced methodological objections, which Lux (2018) described in detail. Overall, there is broad consensus that demand-side, voter-level socioeconomic determinants – especially when operationalized multi-dimensionally – do predict support for populist parties such as the AfD in line with the losers of modernization theory, though the theory cannot offer a mono-causal explanation (Spier, 2010; Gidron and Hall, 2017; Schmitt-Beck, 2017; Goerres et al., 2018; Hambauer and Mays, 2018; Lengfeld, 2018; Lengfeld and Dilger, 2018; Rippl and Seipel, 2018; Tutić and von Hermanni, 2018; Schmitt-Beck et al., 2019; Cohen, 2021; Pesthy et al., 2021; Manow and Schwander, 2022; Ivanov, 2023). To replicate previous findings, the following hypothesis was derived:

*H1a*: Higher scores on socioeconomic variables (economic satisfaction, subjective SES, income) predict decreased AfD vote intention.

#### 1.4.2 East–West differences in socioeconomic variables

During German reunification in 1990, the Treuhandanstalt (Trust Agency) began privatizing roughly 12,000 formerly state-owned East German firms to transition the GDR’s planned economy into the FRG’s market economy. By the time the Treuhand ceased operations in 1994, however, they could mainly sell firms at a loss, resulting in an overall deficit of about €100 million. Of the 4.1 million jobs in companies initially managed by the Treuhand, only about 1.5 million remained in the subsequently privatized firms. The unsuccessful Treuhand policy pushed East Germany into a pronounced economic crisis in the 1990s (BPB, 2020; Kellermann, 2024). Among other consequences, this immediately led to wage inequality, with substantially lower wages in the East than in the West (Dornbusch et al., 1992). Although wages have tended to converge since reunification, East Germans, on average, still earn 27.1% less than their western counterparts (Smolny and Kirbach, 2011; Dickey and Widmaier, 2021). Other objective socioeconomic indicators (e.g., employment) also attest to a worse socioeconomic situation in East Germany until today (Riphahn and Trübswetter, 2012; Conradt, 2015; Patton, 2019; Margarian and Hundt, 2023). On the other hand, research on East–West differences in subjective assessments of one’s socioeconomic situation remains scarce and inconclusive (Pickel and Pickel, 2020, 2023; Diermeier et al., 2024). To replicate and extend the current state of research, the following hypothesis was derived:

*H1b*: East Germans score lower on socioeconomic variables (economic satisfaction, subjective SES, income) than West Germans.

#### 1.4.3 How to explain the gap: socioeconomic variables?

The evidence on whether the East–West gap in AfD support can be explained by demand-side, voter-level socioeconomic variables is fairly consistent. Multiple studies demonstrated that adding socioeconomic covariates to multivariate models did (almost) not alter the effect of an East–West indicator (i.e., a dummy for residence in East versus West Germany) on AfD vote intention or vote choice, regardless of whether the covariates were subjective or objective measures of SES (Goerres et al., 2018; Steiner and Landwehr, 2018; Arzheimer, 2021; Götzel, 2023; Kühling et al., 2025). Manow and Schwander (2022) provided a detailed statistical analysis of status anxiety and found that, although the underlying mechanisms differed across East and West, status anxiety predicted voting for the AfD to a similar degree in both regions and thus did not account for the East–West gap. Contrary findings – suggesting that socioeconomic variables do explain the gap – are rare and, in the case of Hansen and Olsen (2022), show limited interpretability due to large confidence bounds which they attribute to an insufficient number of analyzed constituencies. The broad consensus that socioeconomic variables do not account for East–West differences in AfD support largely relies on election data collected between 2017 and 2021. To replicate previous findings, the following hypothesis will be re-evaluated with updated data from 2024:

*H1c*: Socioeconomic variables (economic satisfaction, subjective SES, income) cannot explain differences in AfD vote intention between East and West Germans.

### 1.5 Psychological framework

#### 1.5.1 Voting for populist parties like the AfD: psychological explanations

Located at the intersection of the socioeconomic and psychological explanatory framework, relative deprivation theory (Runciman, 1966; Pettigrew, 2015) has often been utilized to explain support for populist radical right parties like the AfD (Emslander et al., 2019; Walther and Isemann, 2019; Kruglanski et al., 2021). It holds that the subjective perception of being relatively (economically) deprived in relation to other people or groups, accompanied by negative emotions, can predict support of populist parties like the AfD – rather than the SES per se (Sachweh, 2020; Kruglanski et al., 2021). There is widespread empirical evidence for this assumption (Lux, 2018; Pickel and Pickel, 2020; Rippl and Seipel, 2021; Zollinger and Häusermann, 2024); though a few studies produced contrary findings (Lengfeld, 2017; Hartmann et al., 2022). Within this framework, it was argued that an interaction of frustrated psychological needs, certain narratives and the personal network (3N model, for further information, see Kruglanski et al., 2019) can drive individuals toward endorsement of societally disintegrated micro-identities (Kossowska et al., 2023). From this perspective, the concept of ingroup grievance (i.e., the degree to which people feel that their most important micro-identity is isolated from and treated unfairly by society) can be framed as a feeling of relative deprivation in relation to an outgroup. It could be demonstrated that the feeling of being unfairly disadvantaged by society varies as a function of different micro-identities and can predict AfD vote choice (Zollinger and Häusermann, 2024).

It can be assumed that the feeling of relative deprivation evokes additional negative emotions (e.g., feelings of degradation, humiliation, or belittlement; feelings of shame), which in turn increase susceptibility to populist ideas (Kruglanski et al., 2021; Salmela and von Scheve, 2017, 2018). Within the same framework, psychological needs can serve as explanatory factors for AfD support. It was shown that deprivation of the basic human need of significance can predict populist attitudes (Kruglanski et al., 2021). This is because populist parties like the AfD, for example, continue to promote narratives of a supposed significance loss of German culture due to immigration. According to significance quest theory (Kruglanski et al., 2022), threat of significance loss is a strong driver to preserve significance and thus could lead to support for the AfD and its apparent solutions to the alleged problems causing the threat of loss (Emslander et al., 2019). Similarly, interindividual differences in the fulfillment of other basic psychological needs like the need for closure (Kruglanski et al., 2021), the need for security (Metten and Bayerlein, 2023), and the need for freedom (Jackman and Volpert, 1996) are thought to predict populist attitudes and voting. Evidence on the direct effects of the aforementioned feelings and needs on AfD support is currently lacking. To replicate and extend the current state of research, the following hypothesis was derived:

*H2a*: Higher scores on psychological variables (feelings of relative deprivation, feelings of individual deprivation, psychological needs) predict increased AfD vote intention.

#### 1.5.2 East–West differences in psychological variables

In recent surveys, between 43 and 59% of East German respondents reported feeling like second-class citizens (Faus et al., 2020; Infratest Dimap, 2023). In broad terms of relative deprivation theory, this can be read as an ingroup grievance (i.e., the sense of being treated unfairly by the wider society). By contrast, only 21% of West Germans stated that East Germans are treated as second-class citizens (Faus et al., 2020). A precise East–West comparison is difficult because comparable surveys typically do not ask West Germans whether they themselves feel like second-class citizens. A solution to this is offered by a less psychological and more economic perspective: In 2018, 35% of West Germans reported receiving less than their fair share of the standard of living, compared with 55 to 60% in the East (Pickel and Pickel, 2020). This is in line with the finding that East Germans are twice as likely to feel left behind than their Western counterparts (Reiser, 2024). Additionally, East Germans repeatedly reported elevated levels of negative feelings of individual deprivation compared to West Germans (Faus et al., 2020; Yoder, 2020; Pickel and Pickel, 2023; Vogel, 2023). Assuming that East Germans generally feel more relatively and individually deprived than their Western counterparts, it could be argued that East Germans also display a stronger quest for significance among other psychological needs, though direct evidence on this matter is lacking. To replicate and extend the current state of research, the following hypothesis was derived:

*H2b*: East Germans score higher on psychological variables (feelings of relative deprivation, feelings of individual deprivation, psychological needs) than West Germans.

#### 1.5.3 How to explain the gap: psychological variables?

Direct empirical evidence on the question whether demand-side, voter-level psychological variables account for East–West differences in AfD support is currently lacking. In light of substantial East–West differences in psychological variables as well as their general predictive power on populist voting, the following hypothesis was derived to extend the current state of research:

*H2c*: Psychological variables (feelings of relative deprivation, feelings of individual deprivation, psychological needs) can explain differences in AfD vote intention between East and West Germans.

### 1.6 Cultural framework

#### 1.6.1 Voting for populist parties like the AfD: cultural explanations

Alongside socioeconomic and individual-psychological approaches, support for populist radical right parties such as the AfD is often analyzed from a cultural explanatory framework. Here, “culture” functions as an umbrella term for a wide range of theories and factors used to explain vote intention or vote choice. To reduce complexity, it can be divided into a political-cultural and an ideological-cultural framework.^3^ The political strand centers on a global erosion of democratic culture and mainly draws on the post-democratization theory (Crouch, 2004). In this view, rising globalization and neoliberal marketization have shifted control and problem-solving competency away from nation-states toward supranational institutions and transnational corporate networks. In the eyes of many citizens, national politics appear to be driven more by lobbying and economic interests than by the common good. The results are decline in democratic participation, political disaffection, distance from the political system, and distrust of democratic processes and institutions – dynamics that can foster receptivity to populist radical right ideas and parties (Crouch, 2016, 2023; Rippl and Seipel, 2018).

In line with the concept of post-democracy, vote intention for or vote choice of populist radical right parties like the AfD could be significantly predicted by the perception of lacking possibilities for democratic participation (Rippl and Seipel, 2018, 2021), by dissatisfaction with the current democratic system (Hambauer and Mays, 2018; Lengfeld and Dilger, 2018; Steiner and Landwehr, 2018; Schmitt-Beck et al., 2019; Arzheimer, 2021; Hartmann et al., 2022; Götzel, 2023), by political or institutional distrust (Castanho Silva et al., 2017; Goerres et al., 2018; Steiner and Landwehr, 2018; Christner, 2022; Ivanov, 2023), and by elevated levels of conspiracy mentality as a proxy for perceived democratic incapacity and distrust in state institutions (Castanho Silva et al., 2017; Erisen et al., 2021). In addition, distrust of state institutions and conspiracy mentality are associated with greater consumption of alternative political media (Thorbjørnsrud and Figenschou, 2022; Schwarzenegger, 2023; Brems, 2024). These “represent a proclaimed and/or (self-) perceived corrective, opposing the overall tendency of public discourse emanating from what is perceived as the dominant mainstream media in a given system” (Holt et al., 2019, p. 862). Radical right or extreme right magazines like “Compact,” “Kopp Online” and “Junge Freiheit” are among the most popular alternative political media outlets in Germany (Müller and Schulz, 2021). Estimates for Germany placed occasional users of such media at 7 to 17% and weekly users at 2 to 4% (Klawier, 2024). AfD vote intention and vote choice were shown to predict use of alternative political media (Müller and Schulz, 2021; Müller and Bach, 2023), though evidence on the inverse relationship remains scarce (e.g., Brems, 2024). To replicate and extend the current state of research, the following hypothesis was derived:

*H3a*: Higher scores on political variables (distrust of state institutions, political conspiracy mentality, use of alternative political media) predict increased AfD vote intention.

Within the ideological framework, cultural backlash theory is the most prominent proposition to explain populist support (Norris and Inglehart, 2019). It posits that contemporary populist radical and extreme right parties emerged from a “silent counter-revolution” (Ignazi, 1992) – a reaction to profound cultural change and the reordering of societal value systems accompanying Western democratization (Inglehart, 1977). Such progressive causes include, for example, refugee rights, multiculturalism, political correctness, or gender equality. According to cultural backlash theory, many conservative-minded citizens experience such transformations as threatening, activating an “authoritarian reflex” that ultimately channels support toward populist radical right parties. Individual economic grievances are assumed to increase the likelihood of this reflex, which links the theory to previous frameworks. However, the primary emphasis is on populist and nativist attitudinal patterns that arise as a negative reaction to sociopolitical decisions (Norris and Inglehart, 2019; Jetten and Mols, 2021).

In line with this, support of populist radical right parties such as the AfD could be consistently predicted by populist attitudes such as anti-elitism (Pesthy et al., 2021; Rippl and Seipel, 2021; Christner, 2022; Arzheimer, 2023; Hansen and Olsen, 2024), by nativist attitudes such as the desire for societal homogeneity, the opposition of multiculturalism and the perception of a cultural threat (Rippl and Seipel, 2018; Pesthy et al., 2021; Arzheimer, 2023), and, specifically, by anti-immigrant and nationalist attitudes (Schmitt-Beck, 2017; Goerres et al., 2018; Hambauer and Mays, 2018; Lengfeld and Dilger, 2018; Steiner and Landwehr, 2018; Arzheimer and Berning, 2019; Arzheimer, 2021; Rippl and Seipel, 2021; Hartmann et al., 2022; Götzel, 2023; Ivanov, 2023; Kühling et al., 2025). With three exceptions, the models underlying these and previous results were controlled for and compared with socioeconomic predictors, indicating that cultural factors, net of those controls, exert a stronger influence on voting for populist radical right parties than alternative explanatory approaches (see Inglehart and Norris, 2016). To replicate previous findings, the following hypothesis was derived:

*H4a*: Higher scores on ideological variables (populist/nativist attitudes, national identification, importance of being German) predict increased AfD vote intention.

#### 1.6.2 East–West differences in cultural^4^ variables

Three years after reunification, Minkenberg (1993) identified a “wall after the wall” in political culture between East and West Germany. Roughly 30 years later, Pickel and Pickel (2023) concluded that, while perceptions of democracy’s general legitimacy no longer differ, a “wall in the mind” persists with respect to satisfaction with the current democratic system. East Germans still report markedly lower democratic satisfaction and higher perceived political incapacity, as documented in numerous studies and surveys (Conradt, 2015; Sack, 2016; Lengfeld and Dilger, 2018; Tausendpfund, 2018; Decker et al., 2019, 2020; Patton, 2019; Pickel and Pickel, 2020, 2023; Arzheimer, 2021; Reiser and Reiter, 2023; Vogel, 2023; Reiser, 2024). This pattern is commonly attributed to a widespread sense among East Germans of having been collectively and systematically disadvantaged relative to West Germans (see remarks on relative deprivation above or Pickel and Pickel, 2020, 2023). In line with this, East Germans exhibit significantly lower trust in (state) institutions (Conradt, 2015; Pickel and Pickel, 2020; Braun and Trüdinger, 2023). Moreover, trust in the media is generally lower in the East than in the West, with the exception of social media, where the pattern reverses (Pollak and Krüger, 2024). Direct East–West comparisons in regard to use of alternative political media and conspiracy mentality are currently lacking. To replicate and extend the current state of research, the following hypothesis was derived:

*H3b*: East Germans score higher on political variables (distrust of state institutions, political conspiracy mentality, use of alternative political media) than West Germans.

Within cultural backlash theory, economic grievances are thought to increase the likelihood of an “authoritarian reflex” and, in turn, support of populist radical right parties (see above and Jetten and Mols, 2021; Norris and Inglehart, 2019). As previously shown, East Germans still face disadvantages on several socioeconomic dimensions and report pronounced feelings of relative deprivation compared to West Germans. Alongside other mechanisms further discussed in section 4.1.4, this may help explain the recurring finding that East Germans display stronger populist, nativist, and nationalist attitudes (Pesthy et al., 2021; Arzheimer, 2023; Götzel, 2023), especially including stronger right-wing radical (Decker et al., 2020; Arzheimer, 2023; Kühling et al., 2025), anti-muslim (Kalter and Foroutan, 2021), and anti-immigrant attitudes (Lengfeld and Dilger, 2018; Arzheimer, 2021). A direct East–West comparison in regard to strength and importance of national identification (i.e., German culture, values, beliefs) is currently missing. To replicate and extend the current state of research, the following hypothesis was derived:

*H4b*: East Germans score higher on ideological variables (populist/nativist attitudes, national identification, importance of being German) than West Germans.

#### 1.6.3 How to explain the gap: cultural variables?

Evidence on whether the East–West gap in AfD support can be explained by demand-side, voter-level cultural variables is relatively consistent. Multiple studies demonstrated that the effect of an East–West indicator (i.e., a dummy for residence in East versus West Germany) on AfD vote intention or vote choice could be reduced by adding covariates like dissatisfaction with democracy (Steiner and Landwehr, 2018; Götzel, 2023), institutional distrust (Goerres et al., 2018; Steiner and Landwehr, 2018), nativist or populist attitudes (Pesthy et al., 2021; Arzheimer, 2023; Götzel, 2023), and especially anti-immigrant attitudes (Goerres et al., 2018; Arzheimer, 2021) to multivariate models. At the same time, it should be noted that in some cases, even after introducing the full set of covariates, a significant residual East–West effect on AfD vote choice remained (Götzel, 2023). In others, the East–West effect on AfD vote intention did lose statistical significance after adding covariates, yet the coefficient’s magnitude was largely unchanged and only the standard error increased (Steiner and Landwehr, 2018). Beyond the aforementioned covariates, so far, there is no direct evidence on the question whether cultural variables like conspiracy mentality, alternative political media use, and national identification can account for the East–West gap in AfD support. Moreover, all studies cited in this paragraph were based on election data collected through 2021. To replicate and extend the current state of research, the following hypotheses will be re-evaluated with updated data from 2024:

*H3c*: Political variables (distrust of state institutions, political conspiracy mentality, use of alternative political media) can explain differences in AfD vote intention between East and West Germans.

*H4c*: Ideological variables (populist/nativist attitudes, national identification, importance of being German) can explain differences in AfD vote intention between East and West Germans.

## 2 Methods

### 2.1 Study overview

Study 1 assessed differences between East and West Germans across all explanatory frameworks (i.e., to test hypotheses 1b, 2b, 3b, and 4b). These hypotheses were partly pre-registered in May 2025 (https://osf.io/49dnx/overview). Due to a shift in the research goal from predicting East–West differences in sociopolitical variables to predicting the East–West gap in AfD vote intention, further unregistered hypotheses were later added (i.e., hypothesis 1b and parts of hypotheses 2b, 3b, and 4b) and synthesized with the pre-registered ones based on theoretical considerations regarding the explanatory frameworks (see sections 1.4 to 1.6). This shift also explains why not all hypotheses from this pre-registration were tested. Results of study 1 are reported in section 3.2. Building upon those results as well as on the current state of research regarding explanations for the East–West gap in AfD vote intention, the main hypotheses 1c, 2c, 3c and 4c were pre-registered in August 2025 for study 2 (https://osf.io/6bpqg/overview). As a minor alteration, H2c was later changed from a null hypothesis to an effect hypothesis in order to conservatively test against potential explanatory power of psychological variables (see section 1.5.3). Hypotheses 1a, 2a, 3a and 4a were not pre-registered as they resemble necessary pre-conditions for the main hypotheses and could be consistently derived from the literature.

### 2.2 Data, participants and procedure

Between March and December 2024, a three-wave panel survey was conducted within the scope of a research project (“DigiPatch: Moving from Networked to Patchworked Society”) in five European countries: Germany, Poland, Spain, Sweden, United Kingdom. Since the central outcome, AfD vote intention, is only relevant for the German context and was added in the last wave, the current study solely draws on the German subsample from wave 3 and is thus of cross-sectional nature. For wave 3, data collection took place from November 28 to December 24, 2024. Data was collected online through the survey company Pollster and is securely stored in an institutional repository (Kossowska et al., 2024). The study aimed to gather responses distributed according to stratified quotas based on gender, age, and region of residence to reflect the German population distribution.

The online survey included standardized and validated scales as well as newly developed items covering a wide range of variables relevant to measure the explanatory frameworks described above. The entire questionnaire relied on self-report from participants. Two attention checks were included in the survey to ensure data quality. Participants were required to be of adult age. Ethical approval for the study was obtained beforehand from the Research Ethics Committee at the Institute of Psychology at the Jagiellonian University in Krakow. All participants provided written informed consent, adhering to the principles outlined in the Declaration of Helsinki (BMJ, 1996). The online survey took approximately 30 min to complete.

### 2.3 Measurements

This section provides an overview of the items and scales from the online survey that are relevant to the present hypotheses and were thus used for the analyses.

#### 2.3.1 Living in East or West Germany and AfD vote intention

The region (living in East or West Germany) was measured through the item “Mark the region you live in.”, which included all 16 German states. A binary variable was constructed based on whether the marked state had belonged to the FRG (0, “West Germany”) or the GDR (1, “East Germany”) prior to reunification. Participants living in the state of Berlin were excluded since they could not be assigned to either category (due to the division of Berlin before reunification) and since this solution matched the current population distribution in Germany (see section 3.1). AfD vote intention was measured through the item “Which party would you vote for if a parliamentary election took place next Sunday?”, which included all major German political parties as well as the option “would not vote”. A binary variable was constructed based on whether participants would vote for any other party or would not vote (0) as opposed to participants who would vote for the AfD (1).

#### 2.3.2 Socioeconomic variables

Economic satisfaction (“I am satisfied with my personal economic conditions”.) and subjective SES (ladder classification system based on Adler et al., 2000) were measured through single items on a 7- and 10-point Likert scale, respectively. Monthly net household income was constructed as an ordinal variable with 11 answer options. Together, these items were used as indicators for the latent factor *Socioeconomics* in SEM analyses.

#### 2.3.3 Psychological variables

Participants were asked to indicate their identification with 12 different groups (“micro-identities”; see section 1.5.1 and Kossowska et al., 2023). The chosen micro-identities either challenge widely accepted societal norms (Bréchon and Gonthier, 2017; e.g., individuals critical of official narratives from established authorities, scientists, and the official media) or they reflect values widely recognized and integrated into the societal fabric (Halman et al., 2022; e.g., individuals committed to fighting against inequalities). Three items measured the extent to which participants perceived their most important micro-identity as separated and isolated from society and as being treated unfairly on a 5-point Likert scale. In the pre-registered hypothesis 2c, this construct was labeled “perceived microness of one’s own identity”; elsewhere it has been framed as “ingroup grievance”. However, within the framework of relative deprivation theory, the notion of the ingroup being unfairly disadvantaged relative to society can be read as a feeling of relative deprivation (see section 1.5.1). Hence, the three aforementioned items were used as indicators for the latent factor *Relative Deprivation* in SEM analyses.

Further, three items measured to what extent participants felt individually degraded, humiliated, and belittled over the last month on a 7-point Likert scale (Veldhuis et al., 2014). Together, they were used as indicators for the latent factor *Individual Deprivation* in SEM analyses. Lastly, three basic psychological needs (significance, security, freedom) were measured using three 7-point Likert items each. Examples include “I wish I meant more to other people” (significance; based on Kruglanski et al., 2022), “I wish to feel safer and freer from danger and uncertainty” (security; based on Sheldon et al., 2001), and “I wish I could do things more in line with my preferences” (freedom, based on Sheldon et al., 2001). Together, three items each were used as indicators for the latent factors *Quest for Significance, Need for Security,* and *Need for Freedom* in SEM analyses. In turn, these three factors made up the higher-order latent factor *Psychological Needs*.

#### 2.3.4 Political variables

Distrust of state institutions was measured by the item “I cannot trust state institutions”. Political conspiracy mentality was indicated by a 3-item scale, consistent with prior research (Strömbäck et al., 2024). Two items were adapted from Bruder et al. (2013), e.g., “There are secret organizations that greatly influence political decisions” and one item was adapted from Brotherton et al. (2013), i.e., “I think that the official version of events given by the authorities very often hides the truth”. All four aforementioned items were measured on a 7-point Likert scale and used as indicators for the latent factor *Political Distrust* in SEM analyses. *Alternative Political Media Use* was measured by the item “How often do you typically use political alternative media (e.g., media that have clear political agendas)”. As the scale ranged from “several times a day” to “not at all”, a binary variable was constructed (0: no use; 1: use of alternative media) and included in SEM analyses as a manifest indicator. A distinction between left- and right-wing alternative media was not methodologically feasible. Accordingly, the effect of this variable on AfD vote intention might be underestimated due to the fact that left-wing alternative media consumption is not linked to vote intention (Brems, 2024), let alone intention to vote for a right-wing party like the AfD. However, since the flawed operationalization poses the risk of underestimation rather than overestimation of a hypothesized effect, it was deemed acceptable.

#### 2.3.5 Ideological variables

Basic ideological attitudes are often measured using the GAL-TAN dimensions (green/alternative/libertarian versus traditional/authoritarian/nationalist; originally developed by Hooghe et al., 2002). Within this framework, Shehata et al. (2022) developed items to measure populist and nativist values by three 5-point Likert items. These items, regarding negative attitudes toward refugees and multiculturalism as well as a stance on tougher prison sentences for criminals, were included in the present study and used as indicators for the latent factor *Populist/Nativist Attitudes* in SEM analyses. Further, three 7-point Likert items were derived from the Social Alienation Scale (Bélanger et al., 2019) to inversely measure strength of national identification with Germany, as the derived items covered national rather than social identification (i.e., fitting in well with German values, beliefs, and culture). In addition, endorsement of one of the 12 specified micro-identities (“People for whom being German is very important”) was measured on a 7-point Likert scale (see section 2.3.3). Together, these four items were used as indicators for the latent factor *National Identification* in SEM analyses.

#### 2.3.6 Control variables

The effects on AfD vote intention were controlled for gender (male/female), age (under/over 50 years), migration background (no/first or second generation), employment status (working part-time, student, caregiver or not working/working full-time), educational level (school degree or vocational training/academic training), religion (not religious or other religion/Christian), political orientation (Left–Right Scale from the World Values Survey, see Haerpfer et al., 2022), and size of region (village/town or city). All control variables except migration background and political orientation were originally constructed as ordinal variables and are displayed as such in Table 1.

**Table 1:** Description of sample.

Variable	Total sample *N* = 1,000	West Germany *n* = 776^a^	East Germany *n* = 170^a^	
	*n* (%)	*n* (%)	*n* (%)	*χ*^2^ (*df*)^b^
Vote intention				
Other party or would not vote	853 (85.3)	682 (87.9)	127 (74.7)	18.51*** (1)
Alternative for Germany (AfD)	147 (14.7)	94 (12.1)	43 (25.3)	
Gender				
Male	509 (50.9)	395 (50.9)	84 (49.4)	0.35 (2)
Female	489 (48.9)	380 (49.0)	86 (50.6)	
Non-binary	2 (0.2)	1 (0.1)	0 (0.0)	
Age				
18–35 years	141 (14.1)	111 (14.3)	24 (14.1)	0.42 (3)
36–49 years	191 (19.1)	150 (19.3)	30 (17.6)	
50–65 years	368 (36.8)	279 (36.0)	65 (38.2)	
66–99 years	300 (30.0)	236 (30.4)	51 (30.0)	
Migration background				
No	823 (82.3)	633 (81.6)	150 (88.2)	3.89* (1)
First or second generation^c^	177 (17.7)	143 (18.4)	20 (11.8)	
Employment status				
Working full-time	420 (42.0)	325 (41.9)	72 (42.4)	1.86 (6)
Working part-time	130 (13.0)	101 (13.0)	20 (11.8)	
Stay-at-home parent, student, or other	59 (5.9)	46 (5.9)	9 (5.4)	
Retired	363 (36.3)	283 (36.5)	64 (37.6)	
Unemployed	28 (2.8)	21 (2.7)	5 (2.9)	
Educational level				
Completed secondary school	174 (17.4)	136 (17.5)	27 (15.9)	6.36 (6)
Post-secondary vocational training	109 (10.9)	94 (12.1)	11 (6.5)	
Academic qualification up until bachelors	498 (49.8)	383 (49.4)	91 (53.6)	
Academic qualification up until masters	215 (21.5)	160 (20.6)	40 (23.6)	
PhD or equivalent	4 (0.4)	3 (0.4)	1 (0.6)	
Religion				
Christian	456 (45.6)	419 (54.0)	27 (15.9)	92.11*** (6)
Other	24 (2.4)	20 (2.5)	1 (0.6)	
Not religious	507 (50.7)	326 (42.0)	140 (82.4)	
Decline to answer	13 (1.3)	11 (1.4)	2 (1.2)	
Residential size				
Village	285 (29.2)	242 (31.8)	43 (26.2)	3.32 (3)
Town up to 200,000	413 (42.3)	333 (43.8)	80 (48.8)	
City from 201,000 to 500,000	88 (9.0)	69 (9.1)	19 (11.6)	
City above 500,000	190 (19.5)	116 (15.3)	22 (13.4)	
	M (*SD*)	M (*SD*)	M (*SD*)	*t* (*df*)
**Political orientation (1: left; 10: right)**	4.99 (1.67)	4.99 (1.61)	5.02 (1.83)	−0.23 (230.28)

All decimal numbers were rounded to one or two decimal places. Valid percentages are reported excluding missing cases. No missing cases were observed except for residential size (*n* = 24). ^a^*n* = 54 participants from Berlin were excluded; ^b^ χ ^2^ and t statistics for comparison of variable distributions between West and East Germany; ^c^Either the participant or the parent(s) of the participant were born outside Germany. *p < .05; **p < .01; ***p < .001. Bold values indicate statistical significance.

### 2.4 Statistical analyses

First, descriptive analyses of all variables were conducted. Descriptives of control variables were compared between East and West Germans to present relevant sociodemographic characteristics of the present sample. Then, within the scope of study 1, differences between East and West Germans were assessed for all variables across all explanatory frameworks, using Welch *t*-tests and effect size measures to test hypotheses 1b, 2b, 3b, and 4b.

In the course of study 2, the four central hypotheses 1c, 2c, 3c and 4c as well as their pre-conditional hypotheses 1a, 2a, 3a and 4a were tested using SEM. The central mediation hypotheses contain the term “explain” which is used to denote a statistically significant indirect effect (*a x b*), while the direct effect *c* is primarily used to characterize the type of mediation (Zhao et al., 2010). In a first step, one model was specified for each of the four explanatory frameworks. In each model, the region (living in East or West Germany) was modeled as a single binary exogenous variable; AfD vote intention as the central outcome was modeled as a single binary endogenous variable. Depending on the framework, various latent factors were modeled as mediators and both indirect and direct effects of the exogenous on the endogenous variable were specified. Mediators included the latent factors *Socioeconomics* (model 1: socioeconomic framework), *Relative Deprivation, Individual Deprivation, Psychological Needs* (model 2: psychological framework), *Political Distrust*, complemented by the manifest indicator *Alternative Political Media Use* (model 3: political framework), *Populist/Nativist Attitudes,* and *National Identification* (model 4: ideological framework). In a second step, an overarching model simultaneously integrating all explanatory frameworks was specified (model 5). In this model, latent factors from models 2 and 4 (and the latent factor and manifest indicator from model 3) were combined into higher-order factors, so that each framework was represented by one latent factor: *Socioeconomics, Psychology, Political Culture,* and *Ideological Culture.*

Each SEM only included complete cases without missing values on model-relevant variables (Hughes et al., 2019). Each model was estimated using the mean- and variance-adjusted weighted least squares method (WLSMV) which provides robust test statistics and standard errors for non-normal data containing a binary outcome variable and ordered indicators (DiStefano and Morgan, 2014). For indirect effects, 95% Monte-Carlo confidence intervals (MC CI) were computed based on the distribution of the product, using robust sampling covariance of paths *a* and *b* from the fitted models. When specifying models 1 to 4, only one manifest indicator showed a factor loading ≤ .40 and was subsequently removed to improve model fit. In model 5, this was not repeated when latent factors showed low loadings on a higher-order factor to not exclude substantive areas of interest. Despite two factor loadings < .4, model fit was still excellent for model 5. In each model, latent residual variances of mediators and the outcome as well as covariances between all latent factors were specified (Rosseel, 2012). To ensure unbiased estimates for the effects of region and the mediators on AfD vote intention, all aforementioned control variables were included in each SEM as they might be confounded with the outcome (for most of the controls, this was already demonstrated elsewhere, e.g., Arzheimer and Berning, 2019; Götzel, 2023; Hartmann et al., 2022).

The significance level to interpret effects was universally set at α = 0.05, except for hypothesis 1c which was formulated as a null hypothesis. Here, to conservatively test against the assumption of socioeconomic variables not explaining the East–West gap in AfD vote intention, beta error must be deemed riskier and thus be reduced by increasing the alpha level (Schumm et al., 2013; Lakens et al., 2018).

## 3 Results

### 3.1 Description of sample

*N* = 1,000 adult participants from Germany took part in the DigiPatch online panel survey in wave 3. Of these participants, *n* = 776 (82.0%) lived in West Germany and *n* = 170 (18.0%) lived in East Germany at the time of survey. In this regard, the sample distribution did not significantly differ from the German population distribution (West: 83.3%; East: 16.7%; Foroutan et al., 2023), as shown by a chi-square test [ $\chi^{2}$ (1) = 1.10, *p* = .295]. Table 1 suggests that East and West German subsamples differed significantly regarding vote intention, share of participants with migration background, and religion. Gender and age were evenly distributed and corresponded to total sample distributions. East and West Germans did not differ regarding employment status, educational level, residential size, and basic political orientation.

### 3.2 Study 1 results

For each variable from each of the four frameworks, differences between participants living in East and West Germany were tested. Table 2 presents descriptive data on those variables as well as results of Welch *t*-tests and effect size measures. East and West Germans did not differ regarding economic satisfaction, subjective SES, feelings of individual deprivation, and psychological needs. The monthly net household income was significantly higher among West Germans and East Germans reported stronger feelings of relative deprivation; both effects were of small size. Thus, hypotheses 1b and 2b were supported only in regard to income and relative deprivation and had to be rejected otherwise. On the other hand, East and West Germans seemed to differ across all variables from the cultural (i.e., the political and ideological) explanatory framework. East Germans reported significantly more distrust of state institutions, a stronger political conspiracy mentality, more use of alternative political media, stronger populist/nativist attitudes, and greater strength and importance of national identification than West Germans. These effects were of small to mostly moderate size. Accordingly, hypotheses 3b and 4b were supported.

**Table 2:** Study 1 results: East-West differences across explanatory frameworks.

Variable	Total sample *N* = 1,000	West Germany *n* = 776 ^a^	East Germany *n* = 170 ^a^		
	*M* (SD)	*M* (SD)	*M* (SD)	*t* ^b^	*d*
Socioeconomic variables					
Economic satisfaction (1 to 7)	4.42 (1.77)	4.43 (1.76)	4.34 (1.84)	0.62	0.05
Subjective SES (1 to 10)	5.54 (1.65)	5.59 (1.65)	5.44 (1.62)	1.06	0.09
Monthly net household income (1 to 10)	5.53 (2.53)	5.42 (2.55)	4.90 (2.44)	2.43*	0.21
Psychological variables					
Relative deprivation (1 to 5)	2.65 (0.85)	2.62 (0.85)	2.78 (0.79)	−2.40*	−0.20
Individual deprivation (1 to 7)	1.77 (1.32)	1.77 (1.33)	1.77 (1.35)	−0.01	0.00
Quest for significance (1 to 7)	3.23 (1.61)	3.28 (1.62)	3.05 (1.54)	1.74	0.14
Need for security (1 to 7)	3.86 (1.38)	3.81 (1.37)	3.97 (1.37)	−1.41	−0.12
Need for freedom (1 to 7)	3.60 (1.48)	3.57 (1.46)	3.62 (1.49)	−0.44	−0.04
Political variables					
Distrust of state institutions (1 to 7)	3.42 (1.91)	3.32 (1.88)	3.82 (1.97)	−2.99**	−0.26
Political conspiracy mentality (1 to 7)	3.27 (1.77)	3.15 (1.73)	3.68 (1.84)	−3.36***	−0.30
Alternative media use: see below					
Ideological variables					
Populist/nativist attitudes (1 to 5)	3.61 (0.93)	3.56 (0.93)	3.80 (0.86)	−3.25**	−0.26
National identification (1 to 7)	5.25 (1.20)	5.20 (1.19)	5.45 (1.18)	−2.44*	−0.21
Importance of being German (1 to 7)	3.18 (1.94)	3.09 (1.93)	3.67 (1.95)	−3.55***	−0.30
Use of alternative political media	*n* (%)	*n* (%)	*n* (%)	χ ^2^ (*df*)^b^	
No	461 (46.1)	373 (48.1)	63 (37.1)	6.37* (1)	
Yes	539 (53.9)	403 (51.9)	107 (62.9)		

Higher values indicate greater expression of the corresponding variable. All decimal numbers were rounded to one or two decimal places. Valid percentages are reported excluding missing cases. No missing cases were observed except for household income (*n* = 51) and relative deprivation (*n* = 41). ^a^*n* = 54 participants from Berlin were excluded; ^b^*t* and χ ^2^ statistics for comparison of variable distributions between West and East Germany. *p < .05; **p < .01; ***p < .001. Bold values indicate statistical significance.

### 3.3 Study 2 results

Table 3 presents fit indices for each SEM. Satorra-Bentler scaled chi-square statistics indicated significant misfit for all models (Satorra and Bentler, 1999); however, this likely reflected the test’s sensitivity in large samples rather than poor model fit. As recommended for larger samples, additional fit indices were used to assess overall model quality (Bentler and Bonett, 1980; Berning, 2018). According to standard conventions (Hu and Bentler, 1999) and modern considerations (Niemand and Mai, 2018) regarding fit indices, all models fit the data sufficiently. Therefore, the models were deemed suitable for testing hypotheses 1c to 4c. Initially, only one overall model 5 was planned (see section 2.4). However, in accordance with the theoretical background (see section 1.6), results for model 5a indicated significant overlap of the higher-order latent factors *Political Culture* and *Ideological Culture*, so they were merged to the next higher-order factor *Culture*. This modification did not change model fit of the overall model (see Table 3), but yielded results that were better interpretable in model 5b. Of the control variables, only basic political orientation could significantly predict AfD vote intention across all models (see section 4.1.1).

**Table 3:** Study 2 results: fit indices for structural equation models 1 to 5b.

Model	*n*	*χ* ^2^	*df*	CFI	TLI	RMSEA [90% CI]	SRMR
Model 1 (Socioecon.)	874	168.578***	28	.885	.975	.076 [.065, .087]	.028
Model 2 (Psychol.)	888	698.99***	228	.987	.993	.048 [.044, .052]	.043
Model 3 (Political)	923	292.63***	48	.981	.994	.074 [.066, .083]	.044
Model 4 (Ideological)	923	933.16***	64	.857	.953	.121 [.115, .128]	.074
Model 5a (Overall 1)	842	3175.27***	644	.946	.963	.068 [.066, .071]	.079
Model 5b (Overall 2)	842	3230.37***	647	.944	.963	.069 [.067, .071]	.081

All decimal numbers were rounded to two or three decimal places. Estimation method: mean- and variance adjusted weighted least squares. *χ*^2^scaled chi square statistic based on Satorra and Bentler (1999). df: model degrees of freedom. CFI, Comparative Fit Index. TLI, Tucker-Lewis Index. RMSEA [90% CI]: Root Mean Square Error of Approximation with two-sided 90% confidence interval. SRMR: Standardized Root Mean Square Residual. ***p < .001. Bold values indicate statistical significance.

**Figure 1:**
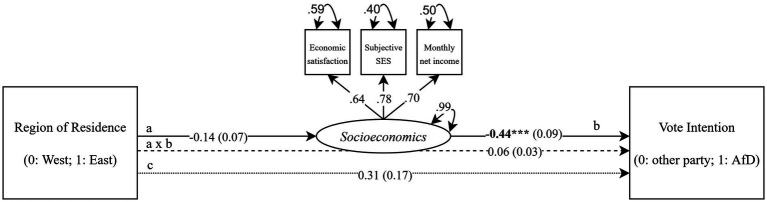
Structural equation model 1: Socioeconomic variables. All decimal numbers were rounded to two decimal places. Based on *n* = 874 cases. The model was controlled for gender, age, migration background, employment status, educational level, religion, political orientation, and size of region. Unstandardized path coefficients (standard error in parentheses), standardized factor loadings, and standardized residual variances are shown. The indirect mediation effect is represented by line a x b. ***p < .001. Bold values indicate statistical significance.

Figure 1 shows that economic satisfaction, subjective SES, and monthly net household income did not significantly differ between East and West Germans (*B* = −0.14, β = − 0.08, *z* = −1.94, *p* = .053). This largely supported the results of study 1 and the rejection of hypothesis 1b (see section 3.2). Model 1 further suggested that respondents in poorer socioeconomic positions were more likely to intend to vote for the AfD (*B* = −0.44, β = − 0.21, *z* = −4.77, *p* < .001), which supported hypothesis 1a. For interpretation of further results of this model, the significance level is elevated to α = .10 (see section 2.4). Against this backdrop, the indirect effect *a x b* of region on vote intention through *Socioeconomics,* which is only barely including zero [*B* = 0.06, 95% MC CI = (−0.0007, 0.13), β = 0.02], must be interpreted as marginally significant. The direct effect *c* was also statistically significant on a 10% level (*B* = 0.31, β = 0.09, *z* = 1.88, *p* = .060). Taken together, this supported the presence of a complementary mediation effect, likely including an omitted mediator in the direct path (see section 4.1.4; Zhao et al., 2010). Hence, the null hypothesis 1c was rejected: Not controlling for other frameworks, socioeconomic variables did mediate the effect of region on vote intention and therefore could explain regional differences in AfD vote intention between East and West Germans, though the effect was only marginal. Practical implications of this effect are discussed in section 4.1.4.

**Figure 2:**
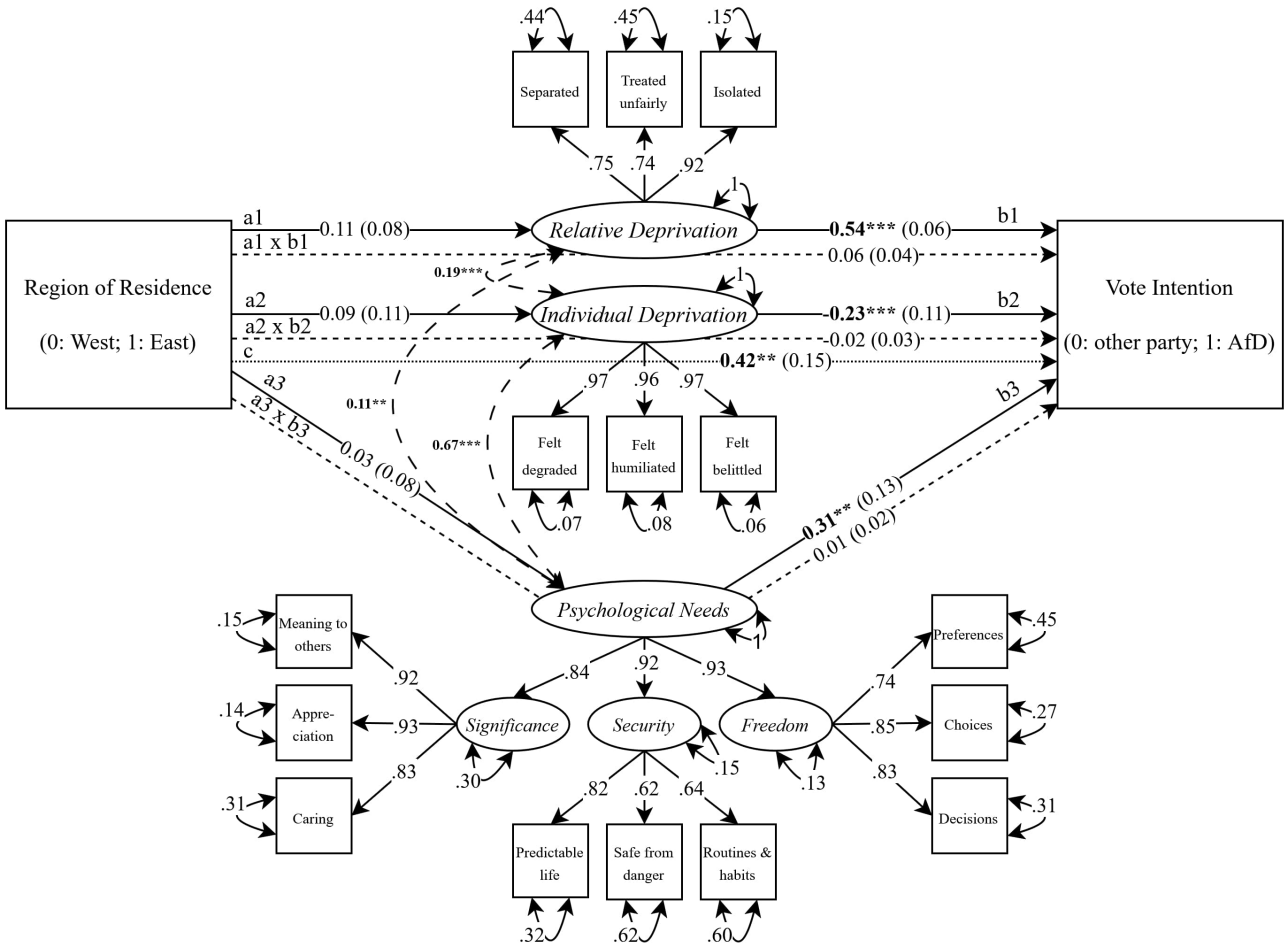
Structural equation model 2: Psychological variables. All decimal numbers were rounded to two decimal places. Based on *n* = 888 cases. The model was controlled for gender, age, migration background, employment status, educational level, religion, political orientation, and size of region. Unstandardized path coefficients (standard error in parentheses), standardized factor loadings, standardized residual variances, and standardized latent covariances are shown. The indirect mediation effects are represented by lines a x b. *p < .05; **p < .01; ***p < .001. Bold values indicate statistical significance.

Figure 2 shows that feelings of relative deprivation (*B* = 0.11, β = 0.06, *z* = 1.40, *p* = .162), feelings of individual deprivation (*B* = 0.09, β = 0.04, *z* = 0.87, *p* = .384), and psychological needs (*B* = 0.03, β = 0.01, *z* = 0.34, *p* = .742) did not significantly differ between East and West Germans. This largely confirmed the results of study 1 and the rejection of hypothesis 2b (see section 3.2). Model 2 further suggested that participants who felt more relatively deprived (*B* = 0.54, β = 0.31, *z* = 8.80, *p* < .001), less individually deprived (*B* = −0.23, β = − 0.17, *z* = −2.03, *p* = .043), and who had higher needs (*B* = 0.31, β = 0.19, *z* = 2.47, *p* = .014), were more likely to intend to vote for the AfD. This only partially supported hypothesis 2a, as the negative association of individual deprivation with vote intention was expected in the opposite direction and is therefore discussed in section 4.1.2. The indirect effects *a x b* of region on vote intention through *Relative Deprivation* [*B* = 0.06, 95% MC CI = (−0.02, 0.14), β = 0.02], through *Individual Deprivation* [*B* = −0.02, 95% MC CI = (−0.09, 0.03), β = − 0.01], and through *Psychological Needs* [*B* = 0.01, 95% MC CI = (−0.04, 0.07), β = 0.00] were not statistically distinguishable from zero. The direct effect *c* was statistically significant (*B* = 0.42, β = 0.13, *z* = 2.81, *p* = .005). Taken together, this supported the absence of a mediation effect through one of the specified mediators (Zhao et al., 2010). Hence, hypothesis 2c was rejected: Psychological variables did not mediate the effects of region on vote intention and therefore could not explain regional differences in AfD vote intention between East and West Germans.

**Figure 3:**
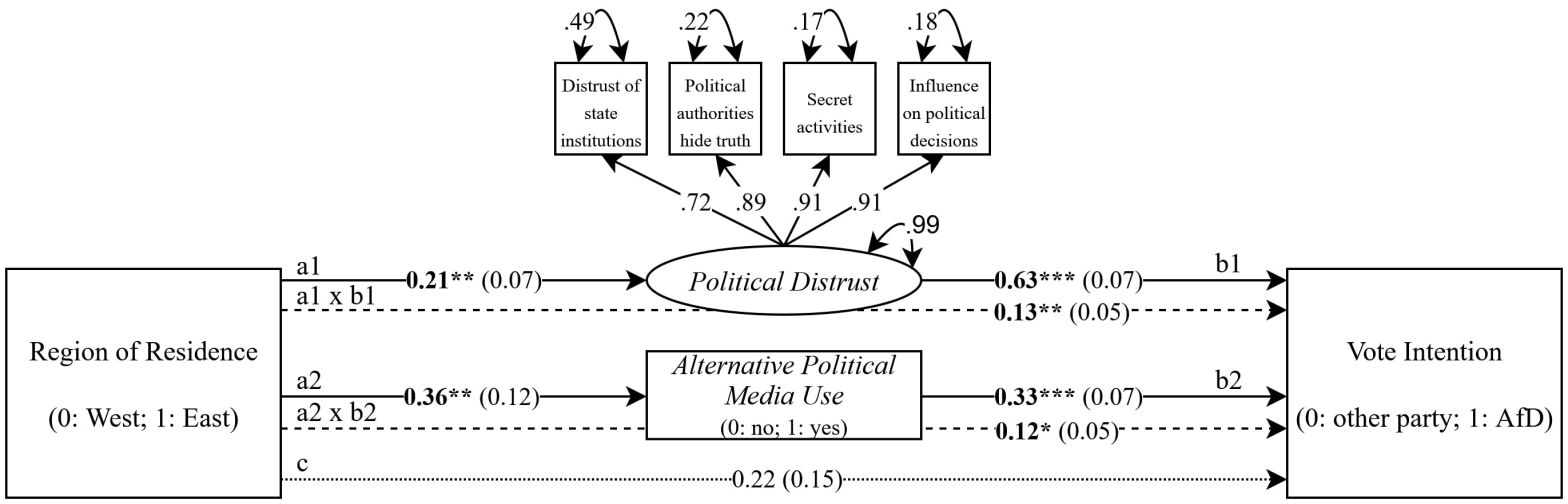
Structural equation model 3: Political variables. All decimal numbers were rounded to two decimal places. Based on *n* = 923 cases. The model was controlled for gender, age, migration background, employment status, educational level, religion, political orientation, and size of region. Unstandardized path coefficients (standard error in parentheses), standardized factor loadings, and standardized residual variances are shown. For reasons of clarity, standardized covariances of alternative media use with “Distrust of state institutions” (.36***), “Political authorities hide truth” (.57***), “Secret activities” (.66***), and “Influence on political decisions” (.61***) are not shown. These were indexed by modification indices > 4 and therefore specified. The indirect mediation effects are represented by lines a x b. *p < .05; **p < .01; ***p < .001. Bold values indicate statistical significance.

Figure 3 shows that East Germans reported significantly higher levels of political distrust (*B* = 0.21, β = 0.11, *z* = 3.10, *p* = .002) and more use of alternative political media (*B* = 0.36, β = 0.14, *z* = 3.10, *p* = .002) than West Germans. This confirmed the results of study 1 (see section 3.2) and the support for hypothesis 3b. Model 3 further suggested that participants who were more politically distrusting (*B* = 0.63, β = 0.34, *z* = 8.74, *p* < .001) and who used alternative political media (*B* = 0.33, β = 0.25, *z* = 4.74, *p* < .001) were more likely to intend to vote for the AfD, which supported hypothesis 3a. The indirect effects *a x b* of region on vote intention through *Political Distrust* [*B* = 0.13, 95% MC CI = (0.05, 0.23), β = 0.04] and through *Alternative Political Media Use* [*B* = 0.12, 95% MC CI = (0.04, 0.22), β = 0.03] were statistically distinguishable from zero. The direct effect *c* was statistically insignificant (*B* = 0.22, β = 0.06, *z* = 1.46, *p* = .144). Taken together, this supported the presence of an indirect-only mediation effect (Zhao et al., 2010). Hence, hypothesis 3c was supported: Political variables did mediate the effects of region on vote intention and therefore could explain regional differences in AfD vote intention between East and West Germans.

**Figure 4:**
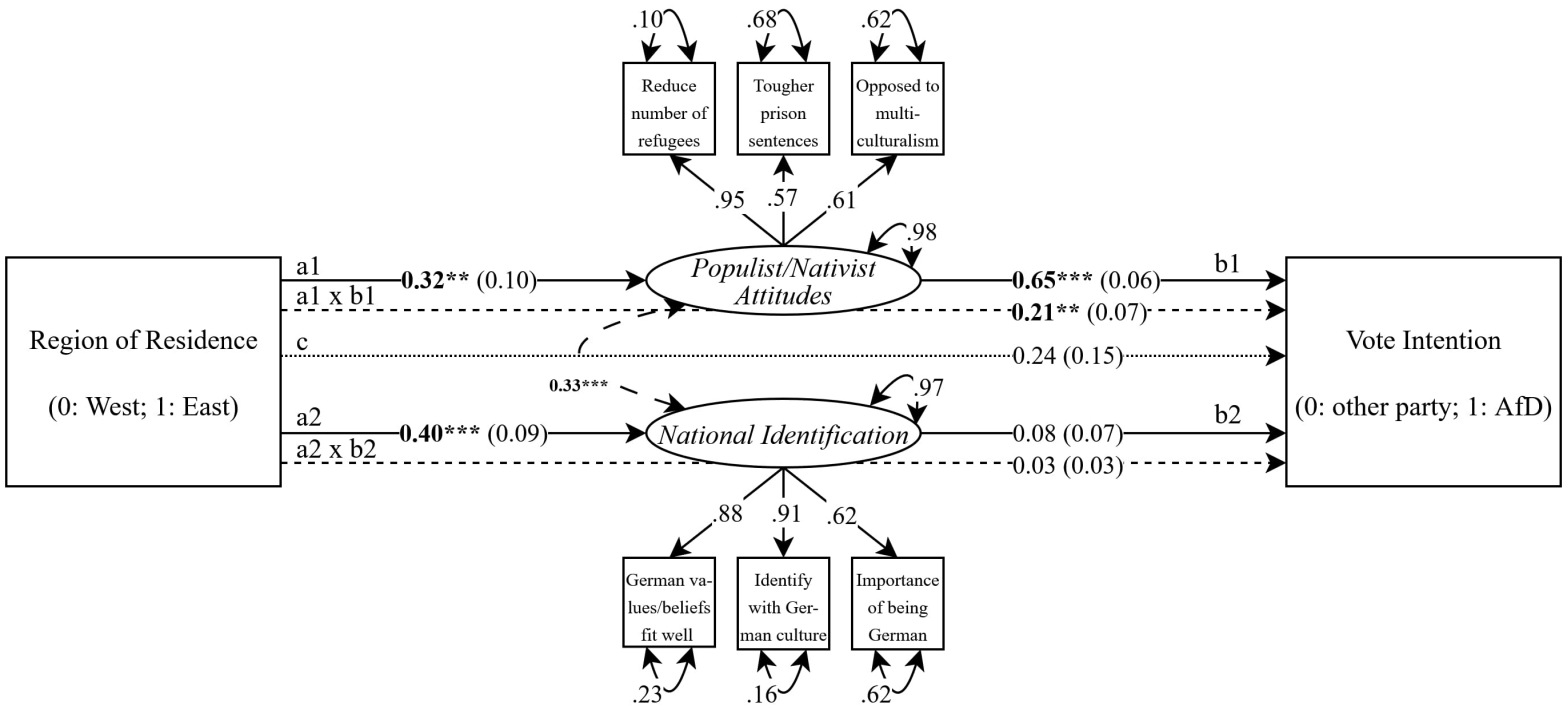
Structural equation model 4: Ideological variables. All decimal numbers were rounded to two decimal places. Based on *n* = 923 cases. The model was controlled for gender, age, migration background, employment status, educational level, religion, political orientation, and size of region. One item from the Social Alienation Scale (“I refuse to be part of German society”; reverse-coded) showed factor loading ≤ 4 and was thus removed from the measurement model for National Identification. This is in line with the other three items measuring national rather than social alienation/identification (i.e., fitting in well with German values, beliefs, and culture, as well as importance of being German). Unstandardized path coefficients (standard error in parentheses), standardized factor loadings, standardized residual variances, and standardized latent covariances are shown. The indirect mediation effects are represented by lines a x b.**p < .01; ***p < .001. Bold values indicate statistical significance.

Figure 4 shows that East Germans reported significantly stronger populist/nativist attitudes (*B* = 0.32, β = 0.13, *z* = 3.17, *p* = .002) and national identification (*B* = 0.40, β = 0.18, *z* = 4.58, *p* < .001) than West Germans. This confirmed the results of study 1 (see section 3.2) and the support for hypothesis 4b. Model 4 further suggested that participants who had stronger populist/nativist attitudes (*B* = 0.65, β = 0.49, *z* = 10.56, *p* < .001) were more likely to intend to vote for the AfD, which supported hypothesis 4a. On the other hand, national identification could not predict AfD vote intention (*B* = 0.08, β = 0.05, *z* = 1.00, *p* = .351), which contradicted hypothesis 4a. The indirect effect *a x b* of region on vote intention through *Populist/Nativist Attitudes* [*B* = 0.21, 95% MC CI = (0.08, 0.34), β = 0.06] was statistically distinguishable from zero, as opposed to the indirect effect through *National Identification* [*B* = 0.03, 95% MC CI = (−0.03, 0.10), β = 0.01]. The direct effect *c* was statistically insignificant (*B* = 0.24, β = 0.07, *z* = 1.58, *p* = .114). Taken together, this supported the presence of an indirect-only mediation effect through *Populist/Nativist Attitudes* and the absence of a mediation effect through *National Identification* (Zhao et al., 2010). Hence, hypothesis 3c was partially supported: Ideological variables partly mediated the effects of region on vote intention and therefore could partly explain regional differences in AfD vote intention between East and West Germans.

**Figure 5:**
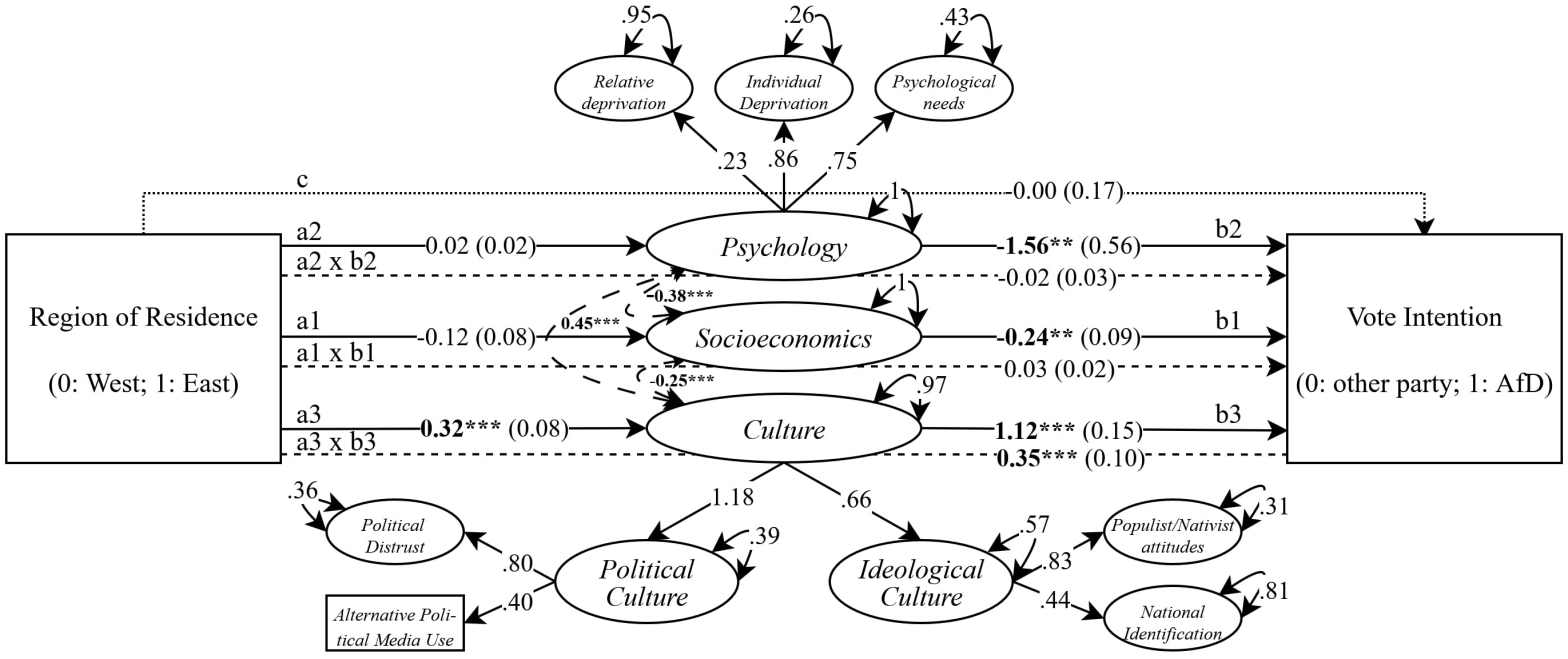
Structural equation model 5b: Overall model 2. All decimal numbers were rounded to two decimal places. Based on *n* = 842 cases. The model was controlled for gender, age, migration background, employment status, educational level, religion, political orientation, and size of region. Unstandardized path coefficients (standard error in parentheses), standardized factor loadings, standardized residual variances, and standardized latent covariances are shown. For reasons of clarity, measurement models of latent factors presented in previous Figures are not shown here again. The indirect mediation effects are represented by lines a x b. * *p* < .05; ** *p* < .01; *** *p* < .001. Bold values indicate statistical significance.

The overall model 5b in Figure 5 once again shows that socioeconomic variables (*B* = −0.12, β = − 0.07, *z* = −1.58, *p* = .114) and psychological variables (*B* = 0.02, β = 0.03, *z* = 0.72, *p* = .475) did not significantly differ between East and West Germans. On the other hand, East Germans reported significantly higher levels on cultural variables than West Germans (*B* = 0.32, β = 0.17, *z =* 4.16, *p* < .001). This confirmed the results of study 1 (see section 3.2). As in previous models, AfD vote intention could be predicted by all explanatory frameworks, though the effect of cultural variables (*B* = 1.12, β = 0.61, *z* = 7.38, *p* < .001) was the strongest when controlled for and compared with the effects of socioeconomic (*B* = −0.24, β = − 0.13, *z* = −2.64, *p* = .008) and psychological (*B* = −1.56, β = − 0.21, *z* = −2.74, *p* = .006) factors. The indirect effect *a x b* of region on vote intention through *Culture* [*B* = 0.35, 95% MC CI = (0.18, 0.56), β = 0.10] was statistically distinguishable from zero, as opposed to the indirect effects through *Socioeconomics* [*B* = 0.03, 95% MC CI = (−0.01, 0.09), β = 0.01] and *Psychology* [*B* = −0.02, 95% MC CI = (−0.10, 0.04), β = − 0.01]. The direct effect *c* was statistically insignificant (*B* = 0.00, β = 0.00, *z* = −0.02, *p* = .985). Taken together, this supported the presence of an indirect-only mediation effect through cultural variables and the absence of mediation effects through socioeconomic and psychological variables (Zhao et al., 2010). The marginal mediation effect of socioeconomic variables (see model 1 in Figure 1) seemed to disappear entirely when controlling for alternative explanatory frameworks within the same model. Hence, in contrast to model 1, model 5b provided some support for the null hypothesis 1c, as well as additional support for hypotheses 3c and 4c. In line with model 2, hypothesis 2c had to be rejected again.

## 4 Discussion

### 4.1 Summary and integration of results

In the present study, three overarching research questions were examined with reference to socioeconomic, psychological, political, and ideological explanatory factors. Specifically, it was assessed to which extent these factors (1) predict AfD vote intention in general, (2) differ between East and West Germans, and (3) account for the East–West gap in AfD vote intention. The key findings addressing these questions are summarized and integrated below.

#### 4.1.1 Descriptive results

Not surprisingly and in line with numerous analyses (e.g., Arzheimer and Berning, 2019; Kühling et al., 2025), an AfD vote intention was more than twice as prevalent among East compared to West Germans in the present sample. Distributions of sociodemographic variables were mostly in line with German census data and other representative surveys. Only the sample distribution of educational levels slightly deviated from the German population, where East Germans are found to be in vocational training more often, while West Germans exhibit more academic degrees (Pfahl et al., 2025). This is considered a minor sampling artifact. Interestingly, despite the significant gap in AfD vote intention, East and West Germans did not differ in their subjective assessment of basic political orientation as indicated by the Left–Right Scale. On average, participants from East and West alike placed themselves in the middle of the political spectrum, likely due to reference group effects. Hence, this variable is irrelevant for explaining the East–West gap in AfD vote intention, as opposed to, for example, differing populist/nativist attitudes, which seem to be independent of the subjective left–right placement to some extent. This ties in with a critical scrutiny of the left–right concept itself (e.g., Jetten and Mols, 2021) and the measurement thereof. Though left–right political orientation as a control variable was a significant predictor of AfD vote intention in all models, meaningful effects of cultural variables remained.

#### 4.1.2 Hypotheses 1a to 4a: how to generally predict AfD vote intention

As predicted and in line with current research (see sections 1.4.1, 1.5.1, 1.6.1), almost all of the included socioeconomic, psychological and cultural variables could meaningfully predict AfD vote intention (see section 3.3). An increased vote intention was associated with a poorer socioeconomic position, stronger feelings of relative deprivation, weaker feelings of individual deprivation, higher psychological needs, more political distrust, use of alternative political media, and stronger populist/nativist attitudes. The only exception was the latent factor *National Identification.* A plausible explanation is that items on identification with German values, beliefs, and culture and on the importance of being German were understood not only in a nativist-nationalist sense, but also as a more civic and liberal identification with the German state and democratic values. This interpretation ties in with national identification showing the smallest East–West difference among all cultural variables (see Table 2), especially against the backdrop of similar East–West approval ratings regarding general legitimacy of democracy (Pickel and Pickel, 2023). It would explain why *National Identification* was not related to AfD vote intention and why it could not explain the East–West gap thereof.

Another notable result is the significant negative association between the latent factor *Individual Deprivation* and AfD vote intention in model 2. Hypothesis 2a contrarily predicted that individual negative emotions would raise AfD vote intention, in line with current research (see section 1.5.1). However, this finding is consistent with other research showing that individual-based deprivation primarily predicts individual-focused outcomes (such as ill-being) and does not reliably translate into political action, whereas group-based deprivation is a robust predictor of collective action and political mobilization (e.g., Smith and Ortiz, 2001; Van Zomeren and Iyer, 2009). This distinction can potentially explain the simultaneous findings of an increased AfD vote intention being positively associated with feelings of relative deprivation and negatively associated with feelings of individual deprivation (see Figure 2).

#### 4.1.3 Hypotheses 1b to 4b: how East and West Germans differ

As predicted and in line with current research (see sections 1.4.2, 1.5.2, 1.6.2), East Germans reported lower income and more relative deprivation, distrust in state institutions, political conspiracy mentality, use of alternative political media, populist/nativist attitudes, national identification, and importance of being German than West Germans (see Table 2). However, hypotheses 1b and 2b were only partially supported, as no differences emerged with regard to economic satisfaction, subjective SES, individual deprivation, and psychological needs. The first two of those findings contrary to hypotheses tie in with studies suggesting that subjective assessments of the personal or regional socioeconomic situation, as opposed to objective indicators such as income or to relative judgments, do not differ between East and Germans (Pickel and Pickel, 2020, 2023; Diermeier et al., 2024). The latter two of those findings are unprecedented, as direct evidence on East–West differences in feelings of individual deprivation and in psychological needs is currently lacking. The absence of such differences in the present sample indicates that the East–West divide might not be a meaningful analytical category for psychological variables like basic individual-based emotions and needs, in line with the recurring finding that core psychological needs are essential for well-being across diverse cultures and regions (Chen et al., 2015).

#### 4.1.4 Main hypotheses 1c to 4c: how to explain the East-West gap in AfD vote intention

Contrary to the null hypothesis 1c, in SEM 1, socioeconomic variables seemed to marginally explain a small fraction of the East–West gap in AfD vote intention. Following the findings in Table 2, it can be plausibly assumed that this was more likely due to differing income levels than to subjective socioeconomic indicators which did not differ between East and West Germans. The marginal explanatory power of *Socioeconomics* contradicts current findings, as the effect of an East–West indicator on AfD vote intention can usually not even be marginally altered by adding (objective) socioeconomic covariates (e.g., Götzel, 2023). However, it should be noted that socioeconomic variables completely lost their explanatory power when adding cultural variables to the same model (see Figure 5), which unambiguously supported hypothesis 1c and ties in with numerous studies producing similar findings (Goerres et al., 2018; Steiner and Landwehr, 2018; Arzheimer, 2021; Pesthy et al., 2021; Götzel, 2023) as well as with the observation that cultural variables better predict populist support in general (see section 1.6.1). Possible reasons for the observed explanatory pattern of socioeconomic variables are discussed below.

Contrary to hypothesis 2c, psychological variables did not explain the East–West gap in AfD vote intention in the present sample, regardless of whether they were analyzed separately (see Figure 2) or controlled for other explanatory frameworks (see Figure 5). This can be mainly attributed to the absence of substantive East–West differences in individual deprivation and psychological needs (see Table 2). The modest difference in relative deprivation (see Table 2) likewise appears to be too small to generate a meaningful mediating effect. Though contrary to prediction, these findings are unprecedented and add to the literature on psychological explanatory mechanisms of the East–West gap in AfD vote intention.

As predicted in hypotheses 3c and 4c, political-cultural and ideological-cultural variables (with the exception of *National Identification*; see section 4.1.2) did explain a substantial share of the East–West Gap in AfD vote intention, regardless of whether they were analyzed separately (see Figures 3, 4) or controlled for other explanatory frameworks (see Figure 5). East Germans averagely exhibited more political distrust, use of alternative political media, and populist/nativist attitudes than West Germans, which in turn increased AfD vote intention among East Germans compared to their Western counterparts. In regard to the examined political-cultural variables, these findings are unprecedented and add to the existing literature. The finding of populist/nativist attitudes meaningfully explaining the East–West gap ties in with the current state of research (see section 1.6.3). Several explanations for this are discussed in the literature. First, in line with realistic conflict theory (Sherif et al., 1961), negative views of minorities or outgroups are attributed to more salient feelings of relative deprivation and perceived competition over scarce economic resources in the East (Mols and Jetten, 2016). Second, the GDR’s state-imposed antifascism, which assigned responsibility for Nazi crimes exclusively to West Germany, is argued to have impeded critical engagement with the legacies of the Nazi regime (e.g., racist sentiments and attitudes that persisted after 1945; Kahane, 2018; Rippl and Seipel, 2021). Third, self-selection effects during post-reunification migration from East to West Germany led to disproportionate out-migration of women, younger people, and the higher-educated – subpopulations in which nativist and populist attitudes are generally less pronounced (Arzheimer, 2023).

Interestingly, several of the theories advanced to explain the East–West gap in cultural variables and thus in AfD vote intention ultimately depend on incorporating socioeconomic accounts. In fact, the debate about drivers of populist radical right support has recently moved beyond a strict economy-culture dichotomy toward asking how the two interact and whether economic variables shape outcomes like populist support indirectly via cultural factors (Gidron and Hall, 2017; Bornschier et al., 2021). There is growing empirical support for this view. For example, the effect of economic insecurity on populist vote choice is attenuated by higher institutional trust (except among the most economically insecure respondents; Ivanov, 2023). Lower political trust and interest as well as preferences for a strong government in present times have been causally linked to negative economic exposure to the Treuhand in the early 1990s (Kellermann, 2024) and to living under a communist regime before reunification (Bondar and Fuchs-Schündeln, 2023). Economic variables have also been identified as determinants of satisfaction with democracy (Tausendpfund, 2018; Patton, 2019; Pickel and Pickel, 2023). Cohen (2021) showed that the positive cross-sectional association between economic risk and AfD voting is fully mediated by populist and nativist attitudes, concluding that socioeconomic and cultural approaches to explain AfD voting should be integrated. This is consistent with cultural backlash theory, which posits that economic grievances heighten the likelihood of an authoritarian reflex to the so-called silent revolution (see section 1.6.1; Norris and Inglehart, 2019).

In this light, the socioeconomic explanatory pattern identified here for the East–West gap in AfD vote intention is best interpreted as initial evidence of an interaction between the socioeconomic and cultural frameworks. Socioeconomic variables on their own have marginal explanatory power for the gap, but once socioeconomic and cultural variables are included jointly, the socioeconomic effects vanish completely, as they might be fully mediated by cultural factors. Accordingly, the four frameworks examined here should not be treated as parallel, cross-sectional blocks; rather, the results point to temporally ordered and partly causal relations among them and with regard to their effect on AfD vote intention. This applies not only to socioeconomic and cultural factors but also to the psychological framework, as it was found, for example, that specific social micro-identities can mediate the effect of economic conditions on party identification (Bornschier et al., 2021). However, psychological accounts remain understudied in the search for explanations of populist radical right support (Salmela and von Scheve, 2017), let alone the German East–West gap therein. Therefore, a more integrative perspective should combine socioeconomic, cultural, and psychological approaches alike. In the present analysis, the particular psychological indicators included could not explain the East–West gap in AfD vote intention, yet other constructs within the same framework might (see section 4.3).

### 4.2 Strengths and limitations

A central strength of this study is the use of SEM. Unlike traditional regression or path analysis, SEM treats key constructs as latent and explicitly models measurement error rather than assuming error-free indicators. This yields more informative estimates than much of the prior literature on AfD vote choice or intention, which has largely relied on regression models (see section 1). Also, existing work has mostly compared socioeconomic with cultural-ideological accounts in explaining AfD vote choice or intention. By contrast, constructs such as conspiracy mentality, alternative political media use, and the psychological variables employed here have been largely omitted and rarely assessed for their explanatory power with respect to the East–West gap. The present study extends the field by drawing comparative inferences about the impact of four different explanatory frameworks on both overall AfD vote intention and the East–West gap thereof, using recent data from late 2024 and thus offering an important update in light of the AfD’s ongoing radicalization (Kühling et al., 2025).

The central limitation of the present study lies in its cross-sectional design, which precludes firm causal inference. Reverse causality is conceivable across all explanatory frameworks, as AfD vote intention, potentially driven by factors outside this analysis, could shape the very explanatory variables considered here. Moreover, while (multiple) mediation models are estimated to decompose the East–West gap into indirect pathways, these decompositions should not be interpreted causally, as identifying causal direct and indirect effects would require strong assumptions (e.g., no unmeasured confounding of the mediated relationships), which are very difficult to test, particularly on cross-sectional data (Daniel et al., 2015). Importantly, this limitation does not diminish the study’s main contribution, as the analyses are intended to decompose the East–West gap in AfD support into explanatory factors from today’s perspective, rather than to identify temporal or causal effects. Relatedly, the cross-sectional prediction of vote intention by ideological attitudes is often criticized as “explaining attitudes with attitudes” (Goerres et al., 2018). It could be argued, however, that vote intention is a multidimensional construct and not reducible to attitudinal content alone, as shown in the present analysis. Here, attitudinal variables are examined alongside other explanatory factors to account for the East–West gap, thereby adding incremental predictive value despite some conceptual overlap of independent and dependent variable in this case.

Further limitations regard the operationalization of the binary variables used in the SEMs. The East–West variable was operationalized using respondents’ current federal state of residence rather than their place of socialization in the former GDR or FRG. This is potentially problematic, as the East–West gap in cultural variables is frequently attributed to socialization experiences specific to East Germany (see section 4.1.4). The present analysis, however, is explicitly concerned with the cross-sectional question of which factors account for the East–West gap in AfD vote intention today, irrespective of age or socialization context. Even if some respondents now living in the West were in fact socialized in the East (the reverse flow is far more unlikely given the continued net migration from East to West; Statistisches Bundesamt, 2025), this would at worst bias estimates conservatively by attenuating East–West differences (on the assumption that East-socialized individuals in the West still respond to cultural items in line with their East German socialization). The outcome variable is based on stated vote intention. It is sometimes argued that measures of vote intention or choice may reflect tactical considerations, and some authors therefore prefer party identification as an indicator of long-term partisan attachment (e.g., Lengfeld and Dilger, 2018). Vote intention, however, holds the advantage of capturing a time-specific assessment of likely electoral behavior in the near future, independent of more stable partisan loyalties and thus allowing for a more topical assessment.

Lastly, it should be critically acknowledged that populist and nativist attitudes were modeled as a single latent factor, although these are conceptually distinct constructs despite some overlap (Mudde, 2004, 2007). This decision was primarily data-driven as the questionnaire did not include enough items for a reliable two-factor specification. Given sufficient factor loadings on the combined factor (see Figure 4) and our aim to reduce complexity by capturing overarching explanatory dimensions of the East–West gap, we deemed a joint factor acceptable. Nevertheless, results relating to this factor should be interpreted and compared cautiously, as alternative conceptualizations treat populism and nativism as separate attitudinal constructs (e. g., Pesthy et al., 2021).

### 4.3 Implications for future research

The preceding sections already point to several avenues for improvement in future research. These include, for example, a more fine-grained operationalization of the latent constructs *National Identification*, *Individual Deprivation* (see section 4.1.2), and *Populist/Nativist Attitudes* (see section 4.2), as well as of the East–West variable and AfD vote intention (see section 4.2). With regard to the still underexplored psychological framework, it would be worthwhile to examine whether the strength of regional identity as East versus West German helps account for differences in AfD support between the two regions. Measuring the extent to which individuals for whom this regional identity constitutes the primary identity feel excluded from, and unfairly treated by, wider society would allow relative deprivation to be captured in a more valid way. On that basis, it could be reasonably expected that relative deprivation does explain part of the East–West gap in AfD vote intention, in contrast to the present analysis, which relied on an operationalization that did not necessarily link perceived relative deprivation directly to an East German identity (see section 2.3.3). In addition, future work should consider a broader set of psychological variables (such as anxiety, anger, need for cognition, moral disengagement, belief in simple solutions, right-wing authoritarianism, and social dominance orientation) as potential explanatory variables for the East–West gap in AfD support, as many of these constructs have been shown to generally predict populist support (Aichholzer and Zandonella, 2016; Erisen et al., 2021; Erisen and Vasilopoulou, 2022; Veit et al., 2024).

In the present study, no hypotheses were proposed regarding a potential East–West moderation of the effect of explanatory factors on AfD vote intention, as “interaction effects between the regional indicator and any of the predictors of radical right voting only rarely reach statistical significance” (Kühling et al., 2025, p.12). However, to account for exceptions and below-significance patterns of more pronounced effects in East Germany, interaction terms should be integrated in mediation-centered SEMs in the future, providing a more nuanced picture of East–West differences in populist support. Next, due to the available data and reasons of complexity, supply-side explanatory factors of populist support could not be considered in the present analyses. Following Mols and Jetten (2020), future studies should integrate both demand- and supply-side factors, as they might interact and reinforce each other regarding their explanatory power of voting for populist radical right parties. Lastly, it was argued that the psychological and cultural explanatory variables might serve as mediators for the effect of socioeconomic variables on AfD vote intention (see section 4.1.4). To test this assumption, future investigations should utilize longitudinal (panel) data, thereby providing evidence for temporally ordered and causal relations among all explanatory frameworks. This would reinforce the scarce longitudinal evidence on this matter which exists to date and which has not yet examined AfD support as the primary outcome variable, let alone the East–West gap thereof (e.g., Bondar and Fuchs-Schündeln, 2023; Kellermann, 2024).

## 5 Conclusion

Despite its limitations, this study advances the understanding of explanatory factors for AfD support and the East–West gap therein. By jointly examining four explanatory frameworks in a comparative design, it offers a comprehensive mapping of demand-side, voter-level explanatory factors behind this gap. Mostly in line with the hypotheses, AfD vote intention is associated with a broad range of socioeconomic, psychological, and political, and ideological factors. However, the East–West gap in AfD vote intention is primarily linked to stronger political distrust, alternative political media use, and populist/nativist attitudes in the East, while socioeconomic and psychological variables seem to play a minor role from a cross-sectional perspective.

These findings have several practical implications. For example, strategies aimed at reducing support for populist radical right parties should focus not only on material grievances, but on rebuilding political trust, fostering critical engagement with alternative political media, and addressing populist and nativist orientations through long-term civic and democratic education. Moreover, the present findings call for closer examination of how current levels of political distrust, alternative political media use, and populist/nativist attitudes may themselves have been shaped over time by socioeconomic and psychological conditions. As populist radical right parties increasingly move into the political mainstream and influence the agendas of so-called centrist parties (Mudde, 2016; Mudde and Kaltwasser, 2018), a nuanced grasp of mechanisms behind populist voting is essential for policymakers and party strategists seeking to respond to their rise without reinforcing the very dynamics that sustain them.

## Data Availability

The dataset presented in this article is not readily available because project-generated data is ongoing. Requests to access the dataset and the analysis code should be directed to hermann.siebel@fu-berlin.de. The codebook and further metadata can be found in an institutional repository (https://doi.org/10.57903/UJ/N8M1GY), where all data is securely stored (Kossowska et al., 2024).

## Footnotes

1 As the present article focuses on explaining the East–West gap in AfD vote intention based on current East–West differences in various variables, theoretical considerations on the origins of such differences between East and West Germans will only be briefly outlined. In part, they are being discussed in more detail in section 4.1.4.

2 The theory of relative deprivation sits at the intersection of the socioeconomic and psychological framework and is therefore discussed below under the latter.

3 This distinction is not categorical, as ideological dispositions are inherently political and political orientations are often ideologically informed. However, for reasons of comparability with other research, the term “political” is hereafter used to exclusively denote variables relating to the perception of the German democratic political culture, whereas the term “ideological” refers to more general value-based worldviews and identity-related orientations. Due to their conceptual overlap, both strands are subsumed under the broader cultural framework.

4 At this point, it is important to stress that “cultural” differences between East and West Germans should not be understood in a culturalist or essentialist sense that attributes specific characteristics to a group of people merely by virtue of this group being from East Germany. Such a reading is reductive and lacks empirical support. Rather, the focus is on historically rooted factors that themselves require explanation– a point taken up and discussed in section 4.1.4.
